# Clinical Lecture on Pneumothorax

**Published:** 1896-04-25

**Authors:** S. H. Habershon

**Affiliations:** Assistant Physician and Pathologist to the Brompton Hospital for Consumption and Diseases of the Chest


### Medical Progress and Hospital Clinics.

[*The Editor will be glad to receive offers of co-operation and contributions from members of the profession. All letters should be addressed to* The Editor, at the Office, 428, Strand, London, W.C.]

### CLINICAL LECTURE ON PNEUMOTHORAX.

By S. H. Habershon, M.D., F.R.C.P., Assistant Physician and Pathologist to the Brompton Hospital for Consumption and Diseases of the Chest.

(*Continued from page* 39.)

### III.—Physical Signs of Pneumothorax.

When air finds its way into the pleural cavity the mechanical conditions are entirely altered. It must be remembered that the lung in its air-tight sac (the pleura) only resists the entrance of air through the bronchial tubes by virtue of its own elastic tension. The lungs collapse during expiration, not only through the pressure of the collapsing chest walls, but also by the rebound of their own elasticity. The forces on each side are equal, and keep the heart and mediastinum in a median position. When air enters the pleura the lung instantly collapses, because its surface is now subjected to a pressure of 15 lbs. to the square inch or nearly so. The elastic tension of the opposite lung, as Dr. Douglas Powell has shown, is instantly exerted; and, having no opposing force, draws over the heart to its own side. It is not, therefore, the pressure of air in the opposite pleural cavity that forces over the heart, because in many cases the opening of the pneumothorax is valvular, and the air in the cavity is of small amount, and the pressure, therefore, less than the full atmospheric pressure. Moreover, as Dr. Powell points out, the shifting of the heart is instantaneous. As air continues to accumulate the pressure may gradually increase, and no doubt still further tends to increase the displacement of the heart. This is a very important point, and a great deal of misapprehension exists among students as to the cause of the displacement of the heart. They think at first that it is the entrance of air which forces the heart over to the other side, but Dr. Douglas Powell, in his short monograph, mentions a great number of experiments which have been performed on animals, and which clearly prove that upon the entrance of air the atmospheric pressure had but a very small part to play in forcing over the heart, and that it was almost entirely the elastic tension of the opposite lung which drew the heart over. I emphasise this by repeating it twice, because I think it is a very important point.

When, therefore, from any cause, air enters the pleural cavity, we expect to find an immovable side of the chest, a note resonant or hyperresonant, a displacement of the heart to the opposite side, and an imperfect entrance of air or an altered respiratory pitch. These are, in fact, the three important physical signs of pneumothorax. When also we find from previous observation either that there has been a dulness from apex consolidation, or a healthy chest, and that these signs are altered, and especially when alarming symptoms have supervened with the change of physical signs, the diagnosis is confirmed.

The physical signs may be divided into these three cardinal signs: (1) Hyperresonance of the chest wall where previously the resonance to percussion had been normal or dull; (2) absence of or feeble breath sounds, or amphoric breathing, *i.e.*, imperfect air entry associated with imperfect movement; (3) displacement of the heart to the opposite side. In addition, there are certain signs which, though not always present, are almost pathognomonic of the affection. They are metallic echo, tinkling, whisper, or cough, the bruit d'airain or bell sound, and succussion splash.

**The Cardinal Signs of Pneumothorax.**—(1) Altered resonance, usually hyperresonance, or tympanitic resonance. When the pneumothorax occurs on the right side of the chest, the earliest sign is the disappearance of the normal liver dulness. In a case of pneumonia which was treated at St. Bartholomew's Hospital, this disappearance was the first thing which drew attention to some grave trouble on the right side. The lung had been very carefully examined on more than one occasion, but one morning after a severe attack of pain it was found that where there was previously a normal liver dulness there was now a tympanitic or resonant note. I think there was also displacement of the heart, but it was the tympanitic note that drew attention to the altered state of affairs. The percussion note is usually that of hyperresonance, but under certain circumstances, described by Walshe as being due to an over-distension of the chest from accumulated air, it is said to assume a semi-dull, toneless, or subresonant note. This must be extremely rare, and I can only say that many of my colleagues to whom I have spoken have never observed it. On the left side the chest becomes hyperresonant or tympanitic, and the heart dulness disappears.

(2) Displacement of the heart. The heart will usually be found displaced to the opposite side. In pneumothorax of the left side the apex may be found near the right nipple; in right pneumothorax the heart may be displaced far to the left, and even to the left axilla. Certain cases, occur, however, in which there is very little displacement, and it is probable that in these cases the heart, or rather the pericardium, is bound down by pleuropericardial adhesions to the chest wall. When there is no displacement the diagnosis is rendered more difficult.

(3) Altered breath sounds. It cannot be too often insisted on that the commonest signs observed are feeble or absent breath sounds, but when the respiratory murmur is audible it usually possesses a high-pitched tone of greater amplitude than normal. Amphoric breathing, cavernous, or broncho-cavernous breathing are frequently heard, the intensity of the sound being probably due to the size of the perforation.

Subsidiary signs not included above are—(*a*) Immobility and sometimes enlargement or distension of the chest, with (in rare instances) flattening of the intercostal spaces; (*b*) absent or somewhat feeble vocal vibration; and (*c*) distant or feeble voice resonance, sometimes of the nature of a distant pectoriloquy.

All the three important signs may be present, and yet the diagnosis may be difficult. We depend in such cases in great measure upon the striking superadded signs that are heard, namely—(*a*) The bruit d'airain, or bell sound, produced by the clashing together of two coins or pieces of metal, one of which is resting on the chest wall. The sound, heard with the stethoscope on listening over any part of the cavity, is that of an anvil struck with a hammer—a metallic ringing sound. A very important point is that the bell sound is not conducted beyond the limits of the cavity, and therefore we are able to map out the exact size of the cavity, and tell with accuracy at what point the lung is adherent. We are familiar with this fact in the case of a dilated stomach, which can be exactly mapped out by the bell sound, and with greater accuracy than by the percussion note. I once had the opportunity of proving this fact, which is not always recognised. This case, which has not yet been published, was one which was admitted to the Friedenheim Hospital with an enlargement of the liver. On percussion it was found that there was a large cavity containing air which extended from the right second rib down considerably below the ensiform cartilage to about half way to the umbilicus, and on the opposite side from the third rib down to the liver. The bell sound could be heard over the whole of this area. When operated upon, an enormous hydatid cyst was found which had generated a large amount of gas. The cavity was of enormous size, and entirely subdiaphragmatic, so large that the forceps, which were about eleven inches long, could be passed upwards into t. It was found that the cavity, as mapped out by the bell sound, was much more sharply defined than was possible by percussion. I was enabled to verify this with the kind aid of the operator, who (with the patient of course under chloroform) introduced his long forceps and measured the internal limits of the cavity, while I, with my coins and stethescope, compared the external area over which the bell sound could be heard. (*b*) The metallic tinkle, a sharp metallic click heard during respiration or with cough. There is no doubt that this is due to the reverberations in the cavity of the sounds produced by a moist râle or bubble. The ordinary clicking or bubbling râle has added to its own vibration those produced in the air-containing cavity, probably harmonics of its own primary note. The metallic tinkle can be heard both when the aperture of the pneumothorax has been closed by adhesion or when it is still open. I have been able to prove this by a post-mortem examination. Metallic tinkling was heard on the day of death, and at the examination the opening was found firmly closed by organised or semi-organised lymph. The sound is said to be produced by the bursting of bubbles or the dropping of liquid in the cavity, but it is also possible that it may be due in some cases to the sound of large liquid râles in a cavity in the lung contiguous to the aperture of the pneumothorax, and conducted across the cavity to the ear applied to the chest-wall. (*c*) The breath sounds, the voice sounds, and the cough may also take on a metallic character, and these sounds are usually described as the metallic echo. In the case of the patient shown this afternoon the sound is so striking as to be suggestive of a flapping of the edge of the orifice in the visceral pleura, acting much in the same manner as the valve of a reed pipe. (*d*) Succussion splash is elicited by shaking the patient, or he may himself hear it on moving or coughing. It is a sound, of course, which is indicative of a combination of air and fluid.

**Differential Diagnosis.**—(1) A large vomica in the lung, especially in the lower lobe or in the anterior and sternal edge of the upper lobe or excavation of the entire lung. On two occasions during the last year or two I have seen this condition mistaken for pneumothorax. In each case the heart was not displaced. (*a*) The diagnosis is rendered more obscure by the fact that in some instances, when a pneumothorax occurs owing to the fixation of the pericardium there is no displacement. In the greater number of instances, when the lung is extensively excavated, the heart becomes either apparently (through uncovering and retraction of the lung) or really (through contraction of the lung) drawn to the excavated side. In these the diagnosis of the cavity is more easy. (*b*) Râles: Usually the presence of liquid râles (large bubbling and creaking sounds) is a sign that there is no pneumothorax, for it is extremely rare to find the conduction of these râles except as metallic tinkes over the cavity of a pneumothorax. In one of my patients, whom you will see presently, this point is illustrated, for it will be seen that, probably owing to the irregular adhesion of certain portions of the pleura, the bubbles in the lung can be heard with great distinctness; but as the end of the stethoscope is made to recede from the adherent patches, the sounds will be heard with less and less distinctness. This fact, that râles may be heard sometimes in cases of pneumothorax, complicates the diagnosis. (*c*) The superadded sounds and all the other signs may be present in the case of a cavity. The lesson to be drawn, however, is that the diagnosis of a pneumothorax, when the heart is not displaced, is exceedingly difficult where it occurs, or is supposed to occur, in a case of advanced phthisis.

(2) Air below the diaphragm: (*a*) Subdiaphragmatic abscess from the perforation of a duodenal ulcer or from a gastric ulcer. The latter case is rare. (*b*) Suppurating hydatid of the liver with formation of gas. The difficulty of diagnosing between these two conditions is obvious. In the Friedenheim case, the notes of which are shown, there was considerable doubt as to whether there was not air both above as well as below the lung. It was, therefore, very difficult and almost impossible to tell whether there was a subdiaphragmatic abscess which had perforated the diaphragm or whether the diaphragm was simply pushed up. I do not know any means by which one could diagnose between these two conditions, but there would be some tympanitic sounds in the epigastric region if the disease was below the diaphragm. The bell sound will be found to extend below the sixth rib, and in all probability a tumour in the abdomen may be present. The history of the case is, therefore, of great importance.

(3) The rare occurrence of a diaphragmatic hernia: The intestines have sometimes been found to protrude through a hernial sac into the thorax. In such a case gurgling or stomach sounds would be heard in the chest.

I shall now proceed with your permission to demonstrate, from the remarkable series of morbid specimens in our museum, the salient points in the pathology of the disease that I have referred to.

